# Evaluation of Left Main Coronary Artery Using Optical Frequency Domain Imaging and Its Pitfalls

**DOI:** 10.1155/2020/4817239

**Published:** 2020-06-12

**Authors:** Vincent Roule, Idir Rebouh, Adrien Lemaitre, Mathieu Bignon, Pierre Ardouin, Rémi Sabatier, Fabien Labombarda, Katrien Blanchart, Farzin Beygui

**Affiliations:** ^1^CHU de Caen Normandie, Service de Cardiologie, Caen 14000, France; ^2^Normandie University, UNICAEN, EA 4650 Signalisation, Électrophysiologie et imagerie des lésions d'ischémie-reperfusion myocardique, Caen 14000, France; ^3^ACTION Study Group, Cardiology Department, Pitié Salpêtrière University Hospital, Paris, France

## Abstract

**Objectives:**

We aimed to assess the quality of optical frequency domain imaging (OFDI) of the left main (LM) arterial wall and describe and analyse potential artefacts in this setting.

**Background:**

OFDI is increasingly used to assess ambiguous lesions and optimize LM percutaneous coronary intervention. However, its ability to provide artefact-free high-quality images of coronary ostia and large segments such as the LM remains uncertain.

**Methods:**

We included 42 consecutive patients who underwent OFDI, including LM imaging. Each OFDI frame was subdivided into four quadrants and analysed. The number of quadrants with artifacts was calculated within the proximal, mid, and distal LM and the first 5 mm of the left anterior descending artery (LAD) and/or left circumflex artery (LCX).

**Results:**

The quadrants analysis showed an overall artifact rate of 8.9%, mostly out-of-field (45.1%) or residual blood (44.7%) artefacts. Most artifacts were located in the proximal LM (18.6%) with a stepwise reduction of artifact rates towards distal segments (mid LM 5.8%; distal LM 3.6%, ostial LAD 2.6%, and ostial LCX 0%; *p* < 0.001). While 20 (48.8%) patients had angiographically visible plaques, OFDI showed plaques in 32 patients (76.2%; *p*=0.007).

**Conclusion:**

OFDI can accurately evaluate the LM and detect and assess angiographically unvisualized atherosclerotic plaques providing accurate assessment of >90% of the quadrants of the LM and the ostia of its bifurcation branches. However, artifacts mainly located in the proximal LM and decreasing distally in a stepwise fashion should be considered in the interpretation of OFDI in this setting.

## 1. Introduction

Accurate assessment of the left main (LM) coronary artery disease is crucial to determine treatment strategies and improve prognosis [1]. Significant LM stenosis accounts for 4.8% of all coronary angiograms and is rarely isolated [2]. The diagnosis of LM disease is based on coronary angiography in routine practice. However, due to a short vessel segment, lack of a reference segment in presence of diffuse atheroma, frequent overlapping daughter branches, and foreshortening [3–5], the accuracy of angiographic determination of LM narrowing may be limited [4]. Percutaneous coronary intervention (PCI) of unprotected LM has been increasingly performed over the last decade [6–8]. LM PCI is a high risk and challenging procedure which requires high precision given the large amount of myocardium at risk [1].

Optical coherence tomography (OCT) provides high image quality offering a unique insight into plaque characterisation and detailed structural information pre- and post-PCI [9]. Initially, OCT was not considered suitable for the assessment of the LM because of the large coronary size and poor blood washing [10, 11]. The recently developed optical frequency domain imaging (OFDI) provides higher acquisition speed and larger field of view compared with prior generation time-domain OCT which may potentially overcome previous limitations. OCT and OFDI are increasingly used to assess ambiguous lesions and guide PCI [12]. Nevertheless, dedicated studies testing the possible pitfalls of OFDI imaging in LM remain scarce, especially in a population without previous LM stenting.

The aim of our study was to assess the quality of ODFI imaging and to describe and analyse its potential artefacts in the assessment of LM arterial wall in coronary artery disease patients with or without detectable angiographic LM lesions.

## 2. Methods

### 2.1. Study Population

We retrospectively included all consecutive patients who underwent OFDI including the whole LM and the ostia of its bifurcation branches in our center between May 2015 and August 2018. Patients with previous LM stenting were excluded.

### 2.2. OFDI Image Analysis

The OFDI procedure (Lunawave®, FastView®, Terumo Europe, Leuven, Belgium) was performed as previously described [13]. Images were analysed offline by 2 investigators (VR and IR) using previously validated criteria for OCT plaque characterisation [14–16]. We analysed the whole LM from the LM ostium or catheter tip to the ostia of its bifurcation branches defined as the first 5 mm of the left anterior descending artery (LAD) and/or left circumflex artery (LCX). LM length was obtained from OFDI longitudinal reconstructions and defined as the distance between the first distal frame of the LM at cross-sectional image and the last proximal LM frame before aorta or catheter visualisation. Proximal, mid, and distal LM were defined, respectively, as the first, the second, and the last third of the whole LM [17]. Reference lumen area and percent area stenosis were calculated as previously described [18]. When atherosclerotic plaque was identified, at least 3 measurements of the intima and media thickness were performed where the plaque was largest. When disappearance of the media was observed, a value of 10 *µ*m (normal value usually observed in our practice) was given to allow calculation of the intima/media ratio.

The feasibility of OFDI assessment was measured in the following regions: proximal LM, mid LM, distal LM, and the ostia of its bifurcation branches [17]. For each region, we analysed all frames. Each frame was subdivided into four quadrants: quadrant 1 = 0–90°, quadrant 2 = 90–180°, quadrant 3 = 180–270°, and quadrant 4 = 270–360°. The number of fully assessable quadrants per frame and consequently quadrants with artifacts were calculated [19]. Artifacts were defined as follows:

- Quadrants out of the field of view: “out-of-screen” loss of image;
- Residual blood: suboptimal vessel flushing causing signal-rich blood swirls in the lumen. Residual blood attenuates the OCT light beam and may defocus the beam if red cell density is high;
- Sew-up or seam artifacts: rapid movement of the artery or the imaging catheter during the acquisition of a single cross-sectional image causing misalignment along the circumference of the image;
- Related to eccentric wire position: when the vessel is large in size and in vessel curvature, the catheter alignment could be noncoaxial, and the resulting image appears elliptical. This makes the measurements inaccurate.

### 2.3. Quantitative Coronary Angiography

Quantitative coronary angiography (QCA) was performed offline (CAAS II, Pie Medical, Maastricht, Netherlands) using validated quantitative methods [20]. The following angiographic data were calculated: vessels reference diameter, percentage diameter stenosis, and LM length.

### 2.4. Statistical Analysis

Continuous and categorical variables were expressed as mean ± standard deviation and numbers of patients and percentages and compared between groups identified by the presence or not of at least one artefact using Student's test or the chi square test, respectively. A *p* value of <0.05 was considered statistically significant. SAS software version 9.4 (SAS Institute, Cary, NC) was used for statistical analysis.

## 3. Results

A total of 42 patients fulfilled the inclusion criteria. LM OFDI was performed to evaluate ambiguous LM lesions in 14 and as a part of a long run for other patients. Baseline clinical and angiographic characteristics are reported in Table 1. Most patients presented with myocardial infarction (*n* = 38; 90.5%) and had single vessel disease (*n* = 26; 61.9%).

**Figure 1:**
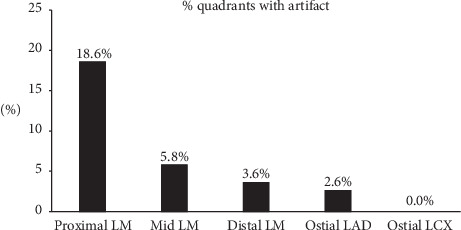
Percentage of quadrants with artifacts in the different explored segments, *p* < 0.0001. LAD, left anterior descending; LCX, left circumflex; LM, left main.

**Figure 2:**
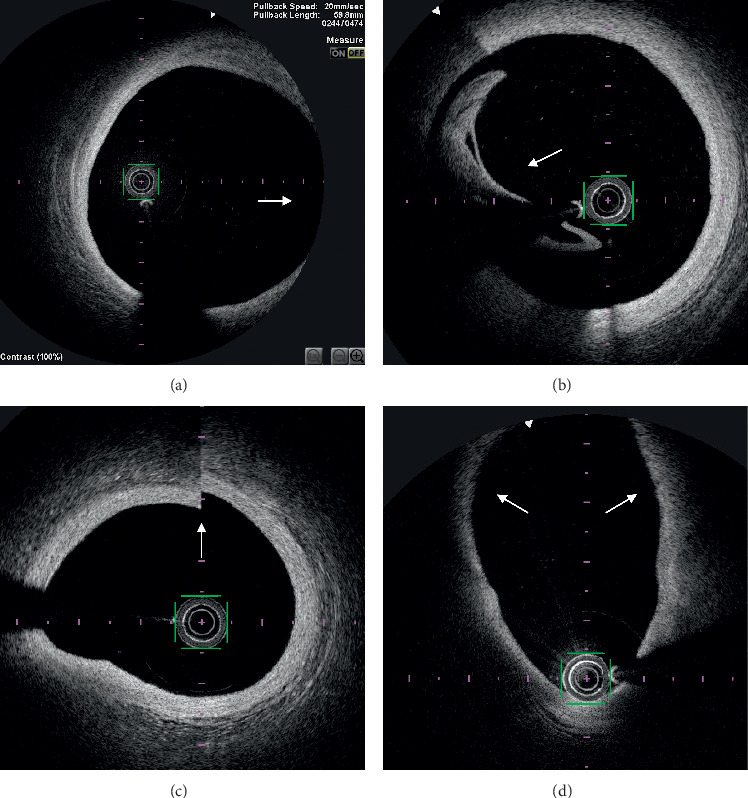
Optical frequency domain imaging examples of artifacts: quadrants out of the field of view (white arrow) (a), incomplete blood displacement by iodine contrast producing volute (white arrow) with high attenuation of optical signal (b), sew-up artifact (c) (white arrow), and artifact related to eccentric wire position causing elliptical image and signal attenuation (d) (white arrows).

OFDI analysis is reported in Table 2. Atherosclerotic disease preferentially affected distal LM (*n* = 13, 40.6%). The quadrants analysis showed an overall artifact rate of 8.9%, which was significantly different across the considered LM segments (*p* < 0.001, Figure 1). Most artifacts were located in the proximal LM (18.6%) and rates decreased distally in a stepwise fashion (mid LM 5.8%; distal LM 3.6%, ostial LAD 2.6%, and ostial LCX 0%). There was a trend towards shorter LM length assessed with OFDI as compared with QCA (10.1 ± 4.5 vs 12 ± 4.38 mm, respectively; *p*=0.0504). Most artifacts (Figure 2) were related to quadrants out of the field of view and residual blood (45.1% and 44.7%, respectively). Other artefacts were sew-up or seam artifacts (7.2%) or related to eccentric wire position (3%). The only variable significantly associated with higher rates of artefacts was the length of LM (11.3 ± 4.8 vs 7.9 ± 2.8 mm; *p*=0.016; Supplementary Table S1). The correlation between artifact's rate and LM diameter was low (*r* = 0.34; *p*=0.026).

**Figure 3:**
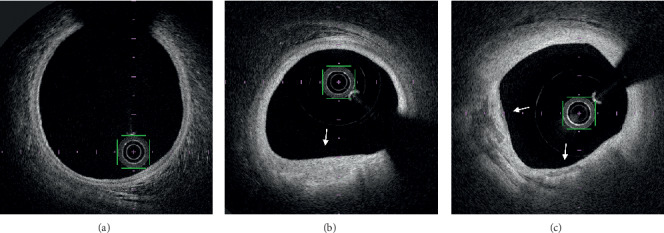
Optical frequency domain imaging examples of plaque analysis: normal LM (a), fibrous plaque (b) (white arrow), and fibrocalcific plaque (c) (white arrows) with intimal hyperplasia and disappearance of the media.

While 20 patients (48.8%) had angiographic signs of LM atherosclerosis, OFDI analysis showed atherosclerosis in 32 patients (76.2%; *p*=0.007). As shown in Table 3, plaques were fibrous (*n* = 22; 68.7%) or fibrocalcific (*n* = 10; 31.3%) and presented intimal hyperplasia (0.53 ± 0.22 vs 0.10 ± 0.02 mm, *p* < 0.001) when compared to normal segments (Figure 3).

## 4. Discussion

Our study showed that overall more than 90% of the quadrants of LM were adequately assessable using OFDI. OFDI detected almost 60% more atherosclerotic plaques than angiography. Artefact rates were low and significantly different between analysed segments, most artifacts being located in the proximal LM. Artefacts were mainly due to quadrants out of the field of view or residual blood effect. When present, plaques were preponderantly fibrous or fibrocalcific with intimal hyperplasia on OFDI analysis.

The angiographic evaluation of LM disease severity can be doubtful or discordant between angiographic views or between operators because of the inherent limitations of angiographic assessment at this site [3–5]. OCT and OFDI are increasingly used to assess doubtful lesions and optimize complex PCI [12]. However, their ability to provide high-quality images in ostial and large calibre segments such as left main coronary artery remains challenged. Our study showed that overall more than 90% of the quadrants of the LM were adequately assessable by OFDI. Previous studies in the setting of pre- or post-PCI have reported poorer performances of OCT in assessing the LM [19, 21]. The most important pitfall reported in one study before PCI was the ability of FD-OCT to fully assess ostial LM in only 12.5% of cases [21]. Parodi et al., who studied LM stenting in 15 patients, reported that 69 ± 20% of the stent inner area (or 2.7 ± 0.8 quadrants/slice) were analyzable, mostly because of quadrants out of screen [19]. The small cohorts and the different OCT systems may participate to the different reported performances. We used OFDI technology which provides a better signal-to-noise ratio, higher acquisition speed during automatic motorized pullback, and larger field of view as compared to latter OCT systems [22]. Additionally, our patients did not have tight stenoses as the OFDI catheter may not cross tight calcified lesions in rare cases.

Most artifacts were located at the proximal part of the LM, as previously reported [21]. This is mostly due to incomplete blood displacement by iodine contrast which is more difficult to obtain for large and proximal segments and quadrants out of the imaging field of view. Other observed image artifacts were sew-up artifacts as the result of rapid artery or imaging system movements during the acquisition of a single cross-sectional image and eccentricity artefact due to the wire position causing elliptical images that attenuate the signal [22]. Nonuniform rotational distortion, fold-over, air bubbles, and saturation artifacts were not observed in our study but may still occur with OFDI.

OFDI provided a more precise evaluation of LM atherosclerotic plaques undetected by angiography in one-third of our patients. OFDI showed mostly fibrous or fibrocalcific plaques with intimal hyperplasia in concordance with previous intravascular ultrasound studies [23, 24]. The lipid core content of LM lesions is less important than in other coronary segments [23, 24] and may explain the greater ability of OFDI to assess the LM wall in absence of attenuation of optical signal beyond the lipid-rich plaques. We found that atherosclerotic disease preferentially affected distal LM encroaching LAD or LCX ostia. Indeed, LM disease is rather diffuse than focal [25, 26] and this point should be considered when treating LM stenosis, as distal disease is associated with worse outcome [27, 28].

Our study showed that OFDI can adequately evaluate LM bifurcation which is the most commonly diseased segment of the LM [26]. On the contrary, operators should consider the limitations of OFDI to assess the ostial LM wall. Tortuous, very large, and short LM may not be ideal for OFDI evaluation. For ostial LM assessment, the choice of the guiding catheter may differ [3]. We preferentially used an extra backup (EBU) guiding catheter which allowed deeper engagement of the LM and consequently adequate contrast flushing. But the true anatomical ostium may have been missed as supported by the shorter LM length observed with OFDI compared to angiography. This is important for optimal stent sizing as malapposition is more common in the proximal than distal LM [29]. The Judkins left (3.5 or 4) guiding catheter can be more easily positioned at the ostium but at the cost of lower flushing and image quality as well as less support in case of further PCI.

### 4.1. Limits

Although larger than most studies on the subject, our study is based on a relatively small sized cohort. We did not assess patients with ostial or very severe LM stenosis which are usually not suitable for OFDI assessment.

## 5. Conclusion

Our study showed that OFDI can accurately evaluate the LM and detect and assess angiographically unvisualized atherosclerotic plaques, providing accurate assessment of >90% of the quadrants of the LM and the ostia of its bifurcation branches. Most artifacts were located in the proximal LM and their rate decreased distally. OFDI can accurately evaluate LM bifurcation, which is the most commonly diseased segment for LM stenosis, and provides a precise evaluation of LM atherosclerotic plaques. However, out-of-field and residual blood-related artifacts should be considered when using OFDI in the ostial or proximal LM.

## Figures and Tables

**Table 1:** Baseline clinical and angiographic characteristics of the study population.

	All (*n* = 42)
Baseline characteristics	
Age (years)	55.6 ± 16
Men	26 (61.9%)
Body mass index (kg/m^2^)	25.9 ± 4.7
Systemic hypertension	15 (35.2%)
Hyperlipidemia	15 (35.2%)
Active smoker	25 (59.5%)
Diabetes mellitus	4 (9.5%)
History of	
Myocardial infarction	4 (9.5%)
CABG	0 (0%)
PCI	5 (11.9%)
Baseline eGFR <60 ml/min	3 (7.1%)
Left ventricular ejection fraction (%)	53.1 ± 10.5

Clinical presentation	
Stable angina	4 (9.5%)
Non-ST-elevation myocardial infarction	10 (23.8%)
ST-elevation myocardial infarction	28 (66.7%)

Angiographic characteristics	
Guiding catheter	
Extra backup	38 (90.5%)
Judkins left guiding catheter	4 (9.5%)
Single-vessel disease	26 (61.9%)
Two-vessel disease	9 (21.4%)
Three-vessel disease	7 (16.7%)
LM length (mm)	12 ± 4.38
LM reference diameter (mm)	4.1 ± 0.56
Proximal LAD reference diameter (mm)	3.1 ± 0.34
Proximal LCX reference diameter (mm)	2.8 ± 0.38
Angiographic signs of LM atherosclerosis	20 (48.8%)
LM stenosis (%) [range]	25 ± 16 [5–70]

CABG = coronary artery bypass graft; eGFR = estimated glomerular filtration rate; LAD = left anterior descending artery; LCX = left circumflex artery; LM = left main; PCI = percutaneous coronary intervention.

**Table 2:** OFDI analysis of the study population.

	All (*n* = 42)
OFDI characteristics	
LM length (mm)	10.1 ± 4.5
LM reference LA (mm^2^)	14.9 ± 4.8
Proximal LAD reference LA (mm^2^)	7.8 ± 3.2
Proximal LCX reference LA (mm^2^)	7.8 ± 4.6
OFDI signs of LM atherosclerosis	32 (76.2%)
LM stenosis (%) [range]	26 ± 18 [7–76]
Tightest lesion site	
Proximal LM	3 (9.4%)
Mid LM	1 (3.1%)
Distal LM	13 (40.6%)
Ostial LAD	11 (34.4%)
Ostial LCX	4 (12.5%)

OFDI imaging analysis	
Global LM analysis	
Quadrants with artifacts/total quadrants	1207/13540
Artifacts (%) [range]	8.9 [0–55.6]
Analyzable quadrants per frame	3.6 ± 0.5
Proximal LM	
Quadrants with artifacts/total quadrants	835/4472
Artifacts (%) [range]	18.6 [0–100]
Analyzable quadrants per frame	3.3 ± 1
Mid LM	
Quadrants with artifacts/total quadrants	223/4516
Artifacts (%) [range]	5.8 [0–62.5]
Analyzable quadrants per frame	3.8 ± 0.5
Distal LM	
Quadrants with artifacts/total quadrants	149/4552
Artifacts (%) [range]	3.6 [0–46.6]
Analyzable quadrants per frame	3.9 ± 0.4
Ostial LAD	
Quadrants with artifacts/total quadrants	172/5800
Artifacts (%) [range]	2.6 [0–47.8]
Analyzable quadrants per frame	3.9 ± 0.4
Ostial LCX	
Quadrants with artifacts/total quadrants	0/1124
Artifacts (%) [range]	0 [0–0]
Analyzable quadrants per frame	0 ± 0

CABG = coronary artery bypass graft; eGFR = estimated glomerular filtration rate; LA = lumen area; LAD = left anterior descending artery; LCX = left circumflex artery; LM = left main; OFDI = optical frequency domain imaging.

**Table 3:** OFDI analysis of the underlying plaque.

OFDI characteristics of the plaque	Diseased LM (*n* = 32)	

Fibrous plaque	22 (68.7%)	
Fibrocalcific plaque	10 (31.3%)	
Cholesterol crystals	5 (15.6%)	
Thrombus	4 (12.5%)	
Microchannels	5 (15.6%)	
Disappearance of media	22 (68.7%)	

OFDI quantitative analysis	Normal LM (*n* = 10)	Diseased LM (*n* = 32)

Intima thickness (mm)^*∗*^	0.10 ± 0.02	0.53 ± 0.22
Media thickness (mm)	0.10 ± 0.02	0.14 ± 0.10
Intima/media ratio^*∗*^	1.0 ± 0.17	4.70 ± 2.51

CABG = coronary artery bypass graft; eGFR = estimated glomerular filtration rate; LA = lumen area; LAD = left anterior descending artery; LCX = left circumflex artery; LM = left main; OFDI = optical frequency domain imaging. ^*∗*^*p* < 0.001 diseased vs normal LM.

## Data Availability

The datasets used and/or analysed during the current study are available from the corresponding author upon reasonable request.

## Supplementary Materials

Supplementary Materials Supplementary Table S1: comparison between patients with and without artefacts on OFDI analysis.
