# Supplementary material for: Evaluation of Left Main Coronary Artery Using Optical Frequency Domain Imaging and Its Pitfalls
Source: J Interv Cardiol. 2020 Jun 12;2020:4817239. doi: 10.1155/2020/4817239 (PMC7306070; doi:10.1155/2020/4817239)
Supplement: Supplementary Materials — Supplementary Table S1: comparison between patients with and without artefacts on OFDI analysis. [file 4817239.f1.docx]

**Supplementary Appendix**

**Table S1:** Comparison between patients with and without artefacts on OFDI analysis

| **Demographic and OFDI characteristics** | **No artefact**  **(n = 14)** | **≥1 artefact**  **(n = 28)** | **p** |
| --- | --- | --- | --- |
| Age | 54 ± 13.92 | 56.4 ± 17.1 | 0.64 |
| Men | 10 (71.4%) | 16 (57.1%) | 0.21 |
| Hypertension | 5 (35.7%) | 10 (35.7%) | 1 |
| Hyperlipidemia | 6 (42.9%) | 9 (32.1%) | 0.49 |
| Active smoker | 11 (78.6%) | 14 (50%) | 0.08 |
| Diabetes mellitus | 2 (14.3%) | 2 (7.1%) | 0.46 |
| Body mass index (kg/m2) | 26.8 ± 5.7 | 25.5 ± 4.1 | 0.40 |
| Left ventricular ejection fraction. % | 53.3 ± 10.5 | 53.4 ± 10.8 | 0.98 |
| Extra Back-Up guiding catheter | 13 (92.9%) | 25 (89.3%) | 0.53 |
| Normal left main | 2 (14.3%) | 8 (28.6%) | 0.31 |
| LM length (mm) | 7.9 ± 2.8 | 11.3 ± 4.8 | 0.016 |
| LM reference LA (mm2) | 14.6 ± 4.5 | 15.3 ± 4.9 | 0.69 |
| LM diameter (mm) | 4.2 ± 0.6 | 4.4 ± 0.6 | 0.23 |
| LM Stenosis (%) | 0.3 ± 0.3 | 0.2 ± 0.1 | 0.52 |
| LM lesion site |  |  |  |
| Proximal LM | 0 (0%) | 3 (10.7%) | 0.20 |
| Median LM | 0 (0%) | 1 (3.6%) | 0.84 |
| Distal LM | 5 (35.7%) | 8 (28.6%) | 0.64 |
| LA=Lumen area ; LM=left main; OFDI=optical frequency domain imaging | | | |
